# Microscopic behavior of nano-water droplets on a silica glass surface

**DOI:** 10.1038/s41598-024-61212-1

**Published:** 2024-05-10

**Authors:** Yuki Araki, Taketoshi Minato, Toyoko Arai

**Affiliations:** 1 Graduate School of Natural Science and Technology, Kanazawa University, Kanazawa, Ishikawa 920-1192 Japan; 2 Institute for Molecular Science, National Institutes of Natural Sciences, Okazaki, Aichi 444-8585 Japan

**Keywords:** Surfaces, interfaces and thin films, Chemical physics

## Abstract

Recent advancements in computational science and interfacial measurements have sparked interest in microscopic water droplets and their diverse behaviors. A previous study using nonlinear spectroscopy revealed the heterogeneous wetting phenomenon of silica glass in response to humidity. Building on this premise, we employed high-resolution atomic force microscopy to investigate the wetting dynamics of silica glass surfaces at various humidity levels. Our observations revealed the spontaneous formation of nano-water droplets at a relative humidity of 50%. In contrast to the conventional model, which predicts the spreading of nanodroplets to form a uniform water film, our findings demonstrate the coexistence of nano-water droplets and the liquid film. Moreover, the mobility of the nano-water droplets suggests their potential in inducing the transport of adsorbates on solid surfaces. These results may contribute to the catalytic function of solid materials.

## Introduction

The adsorption of water from the atmosphere is a continuous phenomenon that occurs across all material surfaces and has been extensively studied in various contexts. For instance, adsorbed water is known to induce detrimental effects, such as rust formation on metal surfaces^1^, degradation of electric devices^2^, and erosion of cement materials^3^. Consequently, efforts have been made toward enhancing water repellency through the microfabrication of metal surfaces and the development of hydrophobic coatings^4,5^.

Water has been historically recognized for its beneficial role as a lubricant on material surfaces and as a protective coating to prevent organic stains on exterior walls and other surfaces^6^. In particular, water adsorbed onto solid surfaces plays a crucial role in enhancing the adsorption of substances within the environmental phase and potentially catalyzing the associated processes. In the tidal flat model, which is a prominent hypothesis concerning the origin of life, inorganic minerals have been suggested to act as catalysts to facilitate the polymerization of amino acid^7,8^. Because minerals that have been identified as potential catalysts, such as clay and carbonate minerals, exhibit hydrophilic properties and tend to adsorb water on their surfaces in moist environments, such as tidal flats, the presence of water on solid surfaces is expected to significantly promote catalytic reactions.

However, there is no clear and complete understanding of the initial process of water adsorption on solid surfaces, because of which the role of water on surfaces and at interfaces is poorly understood. Silica glass, which is a hydrophilic material with a smooth surface, is frequently employed to investigate the wetting behavior of water on solid surfaces. It is widely accepted that on a hydrophilic solid surface, water is uniformly adsorbed, forming a water film comprising several molecular layers.

Notably, investigations employing interface-selective measurement techniques, such as sum frequency generation (SFG) spectroscopy^9^ and frequency modulation atomic force microscopy (FM-AFM)^10^, for mica surfaces have demonstrated the formation of ice-like water films under high-humidity conditions. These films exhibit homogeneous wetting because of the presence of surface OH groups. Studies conducted on a silica glass, with a contact angle resembling that of mica, using attenuated total reflection infrared spectroscopy (ATR-IR), have revealed the presence of approximately two layers of water film, even at a relative humidity (RH) of 20%^11^. Furthermore, the film thickened uniformly in response to increasing humidity in the direction perpendicular to the surface.

In contrast, the application of heterodyne SFG (HD-SFG) analysis, which can help distinguish water molecules at the solid–liquid and gas–liquid interfaces, introduced a model suggesting the heterogeneous wetting of a silica glass surface as the RH increased from 20 to 90%^12,13^. According to this model, water droplets gradually enlarge and ultimately coalesce to form a water film. This phenomenon is attributed to the chemical and structural heterogeneities inherent to the silica glass surface. While vibrational SFG spectroscopy studies have indicated that a glass surface remains incompletely wetted even at a humidity of 90% through wetting measurements^14^, there is no consensus on the heterogeneous wetting of glass surfaces due to discrepancies identified when compared with prior findings.

To address these disparities, this study aimed to visualize the microscopic behavior of water on a silica glass surface. The phenomenon of surface wetting was observed in situ using FM-AFM in a humid environment. Remarkably, our observations suggest the concurrent development of a homogeneous liquid film and nanodroplets on the silica glass surface. Hence, we further evaluated the adhesion force of each phase using peak-force tapping AFM (PFT-AFM) to clarify the differences in the viscosity of them.

## Methods

### Materials and chemical cleaning

We used a 10 mm square quartz glass (IR-grade fused silica FQIR10SQ-1: Pier Optics), which is the same type of sample used in previous spectroscopic research^12^. The surface of the glass was wiped with ethanol (purity: 99.5%, Wako) and then immersed in piranha solution for 10 min. Subsequently, it was cleaned using a UV-ozone generator (NL-UV253, Filgen, Inc.) for 30 min before conducting the experiment to remove surface contaminants. We repeated the UV-ozone cleaning for 1 h each time during the observation, totaling 4 h to verify that the nanodroplets observed were not organic contaminants. The sample was fixed at the center of the open liquid cell^15^, and 200 µL of water was added to the moat around the sample to increase the humidity.

### High-RH experiment by frequency-modulation atomic force microscopy

A modified commercial FM-AFM (prototype SPM-8000FM, Shimadzu Corp.) was used to observe the surface topography. A gold-coated silicon cantilever (ppp-NCH AuD, Nanosensors) with a spring constant of 30–50 N/m and a resonance frequency between 280 and 310 kHz was used. The tip surface is expected to undergo natural oxidation and appear hydrophilic since no special treatment has been applied to the tip. The FM-AFM instrument was placed in an incubator (CN-40A, Mitsubishi Electric Engineering) set to 25 ℃, and the humidity was enhanced by placing a 200 mL cup of water inside the incubator. The relative humidity (RH) in the incubator increased to 80% within 90 min after the door was closed, and it reached equilibrium at nearly 85% (Figure S1). A humidity sensor (HT-3007SD, SATO TECH) was positioned approximately 50 mm from the sample.

### Peak force tapping microscopy

The peak force tapping mode was used for the quantitative measurement of the physical properties. In this study, an atomic force microscope (Dimension XR Icon NanoElectrical, Bruker) operating in the peak force tapping mode was used to measure the adhesion forces on the quartz glass. The adhesion force was obtained from the force curve when the probe is retracted from the sample surface (retract curve). The force was calculated, as in contact mode AFM, by converting the deflection of the cantilever into force using Hooke’s law.^16^ The maximum force applied to the probe during retraction represents the adhesion force (Figure S2). Note that the attractive force is shown as a negative value, with larger absolute value indicating larger adhesion force. The sample used for the FM-AFM measurements was placed in a closed cell (Local environmental cell, Bruker) (Figure S3). The force curves were acquired at 128 × 128 points within the scan area to generate adhesion maps. A silicon nitride cantilever (ScanAsist Air, Bruker) with a spring constant in the range of 0.2–1 N/m was used. The observations were made at room temperature, and the humidity was controlled from 0 to 85% by regulating humidified air (AHCU-2, KITZ micro filter Corp.).

## Results and discussion

### In situ observation of wetting process on silica glass surface by FM-AFM

**Figure 1:**
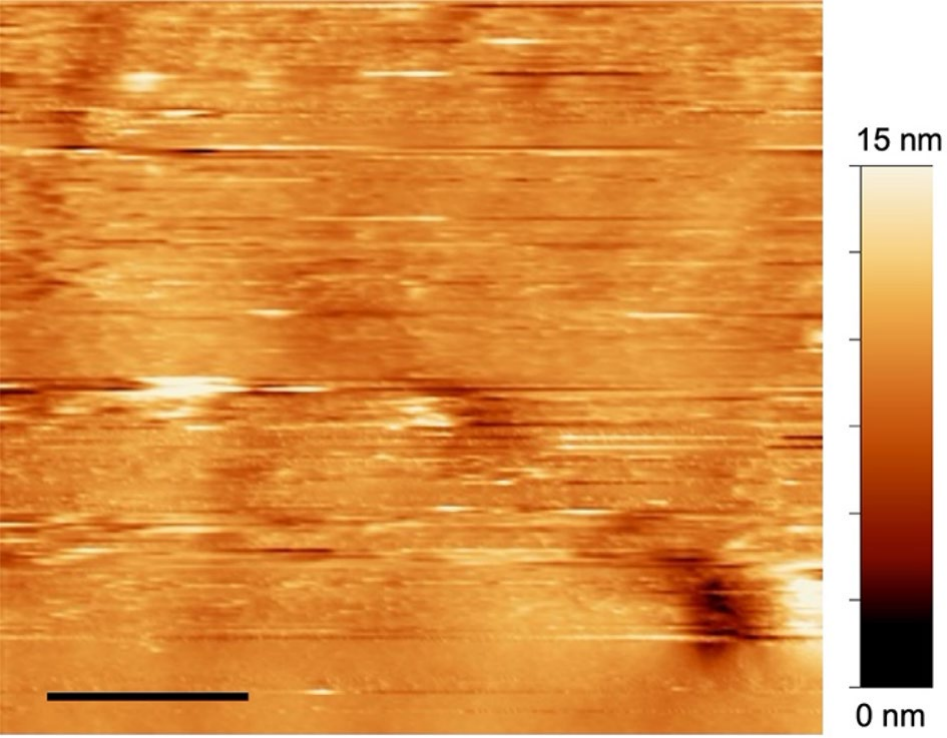
Topographic image of a silica glass surface in dry air (RH 30%) observed by FM–AFM. The scale bar represents 500 nm.

**Figure 2:**
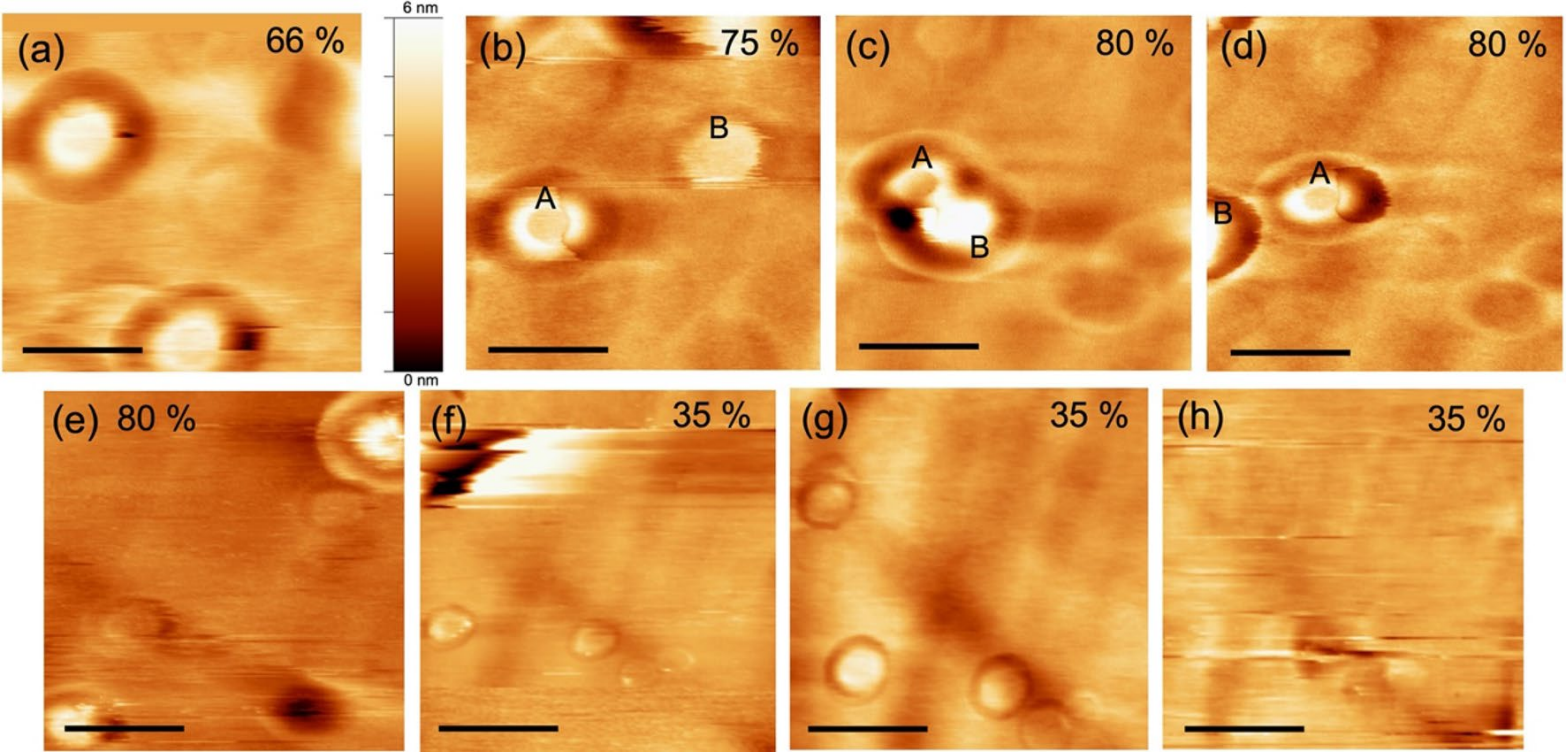
Snapshots of change in the topography of the silica glass surface with humidity in the same scan area by FM-AFM. The upper images were obtained while increasing humidity, and the bottom images were obtained during the process of decreasing humidity. (**b**)–(**d**) depict contact and repulsion between nano-adsorbents. A and B denote the respective nano-adsorbents. The numbers on upper right represent relative humidity. Color bar indicates the Z scale of the images. Set point of frequency shift (*Δf*) and amplitude were 300 ~ 400 Hz and 0.4 nm_p-p_, respectively. All scale bars represent 500 nm.

In dry air at 30% RH, the silica glass surface appeared almost smooth, with an average roughness of 0.95 nm calculated from the height profile along the fast scan direction (Fig. 1). When the humidity increased to 50% RH, circular adsorbates with a diameter of approximately 500 nm were observed on the glass surface. These adsorbates exhibited consistent reproducibility even after repeated photochemical cleaning of the glass substrates. Furthermore, continuous in situ observation of the same area at a scan rate of 2 min per image (Figure S4) consistently revealed one or two adsorbates within an area of 1.5 × 1.5 μm^2^ at RH values ranging from 50 to 80% (Fig. 2a–d). The displacement of the polishing marks on the glass surface by a few ten nanometers confirmed the occurrence of thermal drift during scanning. Nevertheless, the alteration in the relative positions of the adsorbates indicated their diffusion across the glass surface. Upon opening the incubator and swiftly reducing the humidity to 30%, we noted that these adsorbates underwent rapid size reduction and completely disappeared within 15 min of incubator exposure (Fig. 2e–h).

Despite the observed diffusion behavior, the number density of the adsorbates within the scan area remained nearly constant at humidities above 50%, implying a homogeneous distribution of the adsorbates on the glass surface. Furthermore, there was repulsion between the adsorbates (Fig. 2b–d). As shown in Fig. 2b–d, Adsorbates A and B gradually moved closer, made contact, and then moved apart. As shown in Fig. 2c, the overlap between Adsorbates A and B is noticeable on the same scan line, confirming their contact. The height of Adsorbate A changed from 2.5 nm to 1.9 nm, while that of Adsorbate B varied from 1.0 nm to 6.9 nm before and after contact, indicating a mass transfer resulting from their contact. When the adsorbates were surrounded, consistent grooves were observed (further details are provided below).

The height of the adsorbate was measured as the difference between the height of the smooth surface outside the grooves and the apex of the adsorbate. At 50% RH, most of the adsorbates measured a few nanometers in height (Figure S5), whereas one of them expanded to a height of approximately 10 nm at a humidity of approximately 80%. However, in these instances, no alteration in the lateral size was evident. This was attributed to the vertical growth through contact and separation with the other adsorbates, as shown in Fig. 2b–d. Based on their stability exclusively in humid conditions and the fluid nature of materials among the adsorbates, we concluded that these adsorbates were adsorbed water, which we named “nano-water droplets”. They exhibit growth in height on the order of nanometers with increasing humidity, maintaining diameters in a few hundred of nanometers in height and remain stable under high humidity conditions but disappear at around 30% RH. As previously mentioned, these nanoscale water droplets maintained their individuality upon contact without coalescing and exhibited no lateral size alterations, distinguishing them from macroscopic droplets and indicating distinctive properties inherent to their nanoscale structure.

### Behavior of nano-water droplets on a silica glass surface under equilibrium relative humidity

**Figure 3:**
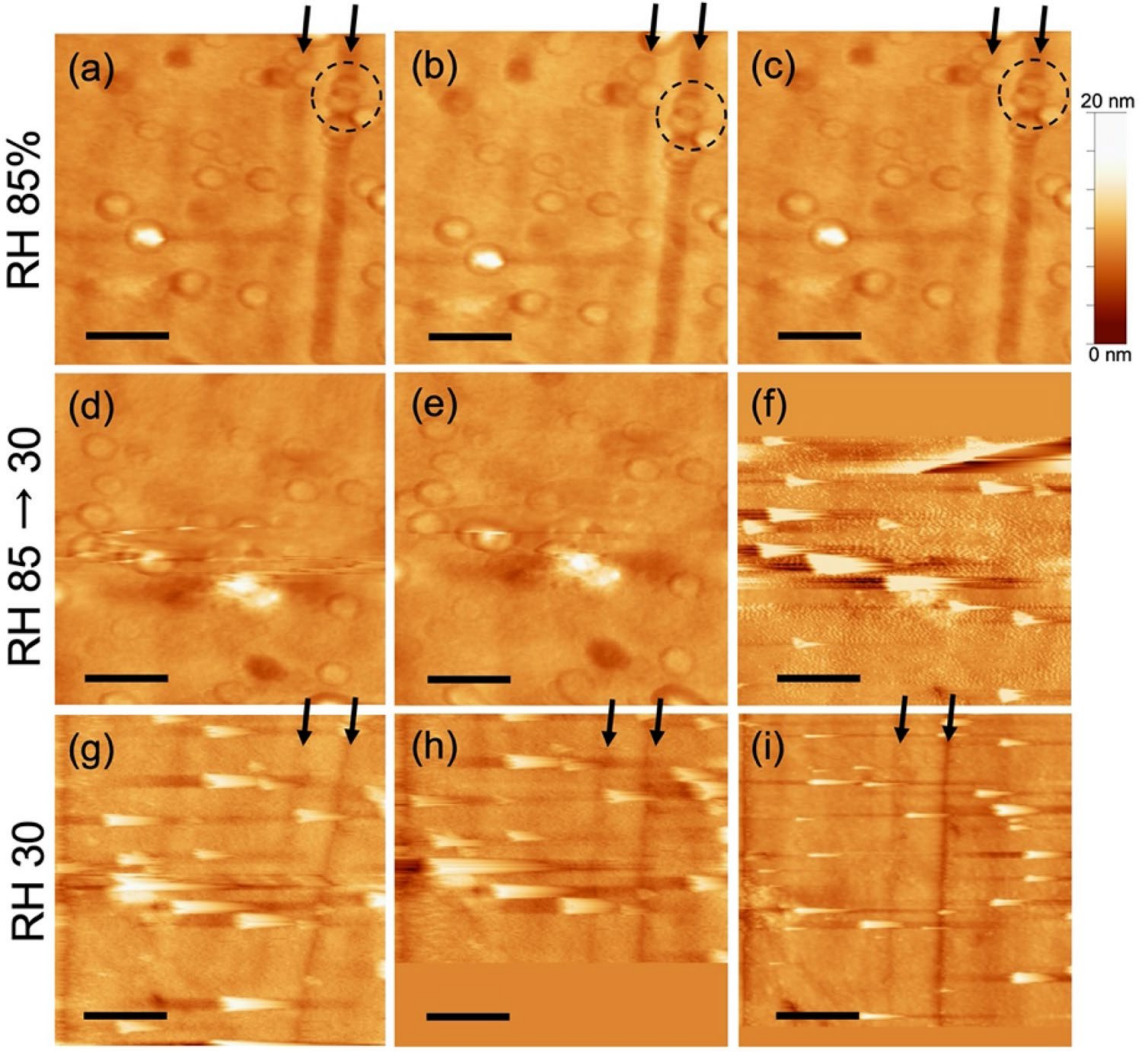
The surface topography of the glass surface left overnight at RH 85%. The sequential images at RH 85% (**a**–**c**), in drying from RH 85% to 30% (**d**–**f**), and after 15 min of drying at RH 30% (**g**–**i**). Within each row, the images progress from left to right. Each image was obtained in 2 min per image. Arrows in the figures indicate the positions of polishing marks on the glass surface, serving as landmarks. It is conjectured that the scan area has shifted due to thermal drift caused by rapid drying in (**d**)–(**f**). Dashed circles in (**a**)–(**c**) represent overlapping droplets. The areas with uniform contrast at the top of (**f**) and the bottom of (**h**) are regions without scan data. Color bar indicates the Z scale of the images. All scale bars represent 500 nm.

The same silica glass was subjected to 85% RH in an incubator overnight for observation. At this time, the nano-water droplets appeared to have an even size; however, their diameter was approximately 200 nm smaller than that observed during the increase in humidity, as detailed in previous section (Fig. 3). Correspondingly, the height of the nano-water droplets was consistent with that described in previous section, with the majority measuring less than 2 nm, whereas rarely reached up to 10 nm (Figure S6). A significant difference from the findings recorded in previous section is the nearly threefold increase in the number of droplets observed within the 1.5 × 1.5 μm^2^ area, with a near absence of surface diffusion of the droplets (Fig. 3a–c). Notably, a few nano-water droplets measuring 2 nm in height exhibited an overlap (circles in Fig. 3). Unlike the contact between the droplets shown in Fig. 2, this overlap is likely an artifact. The contours of each of the two droplets remained distinct, and the overlapping area was smaller than that of the original droplet, rather than being elevated. This phenomenon was observed exclusively within the polishing traces on the glass surface at a depth of approximately 3 nm. Upon opening the incubator and swiftly reducing the humidity to 30%, we found that all the droplets disappeared from the glass surface, similar to that described in previous section (Fig. 3d–f).

However, unlike the results obtained during the increase in humidity, irregularly shaped impurities with a height of approximately 10 nm could be observed adhering to the glass surface at locations where some of the nano-water droplets had formed (Fig. 3g–i). Remarkably, these impurities remained firmly attached to the glass surface, even after the humidity had decreased to 30%. Nano-water droplets lacking surface diffusion were inferred to be heterogeneously nucleated against these impurities. The underlying driving force for the surface diffusion of the nano-water droplets remains unclear. It is unlikely that the probe influenced this phenomenon because the diffusion direction remained independent of the scan direction observed in previous section (Figure S7). Similarly, thermodynamic heterogeneity, such as the vapor pressure near the glass surface, seems to be an improbable cause, because the nano-water droplets exhibited no movement during the drying process in either condition: during the increase in humidity or during overnight exposure to high humidity. The absence of surface diffusion of the nano-water droplets is attributed to the static presence of impurities that acted as nucleation sites for condensation. The number of nano-water droplets was presumed to be influenced by the number of impurities adsorbed onto the glass surface. In contrast, as described in previous section, no impurities were observed following the drying process, indicating that not all the nano-water droplets were formed through heterogeneous nucleation facilitated by impurities.

### Coexistence of nano-water droplets and nano-liquid film on a silica glass surface

**Figure 4:**
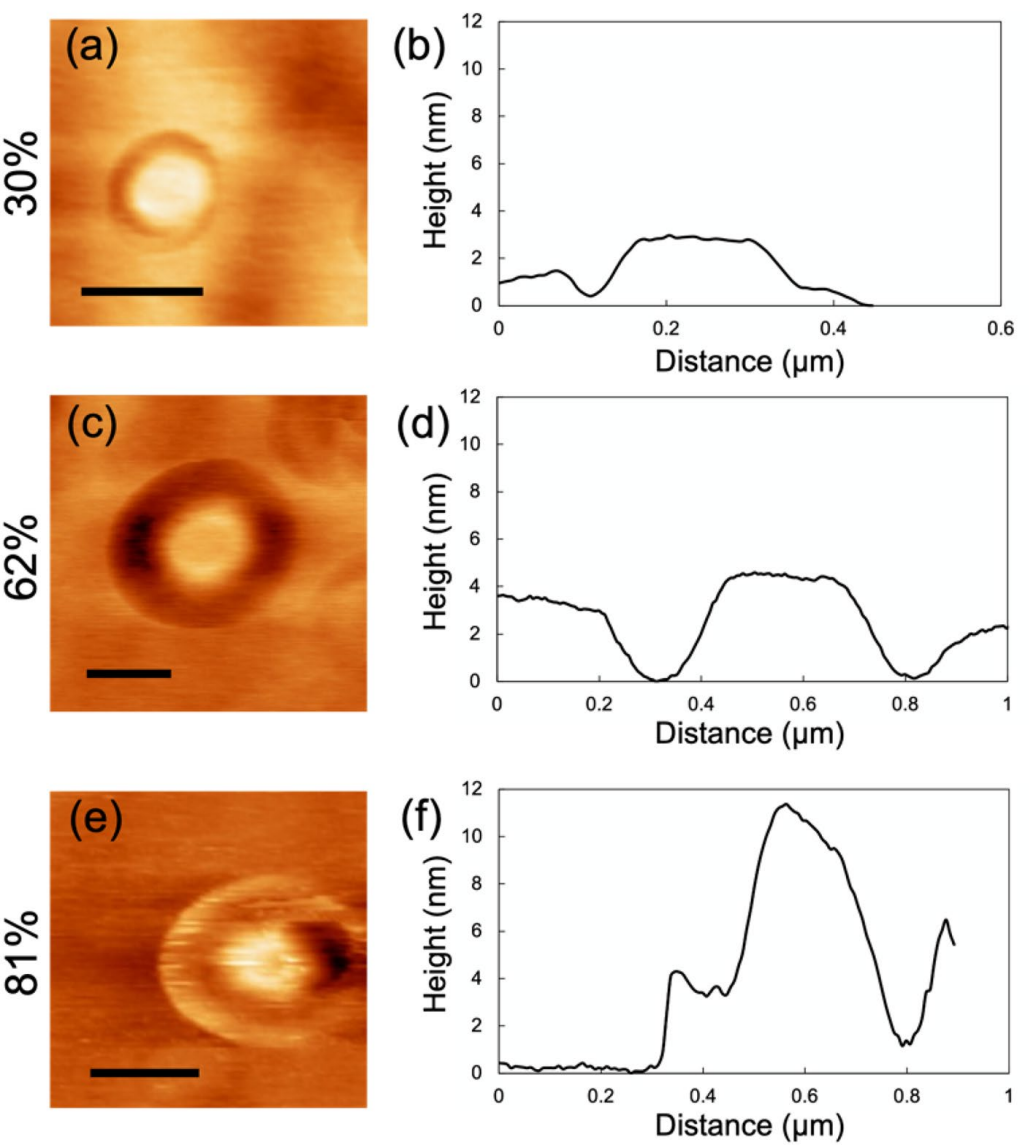
Change in the morphology of the nano-water droplets with the humidity. The images on the left are the surface topographies, and the images on the right are the cross-sectional profiles of the nano-water droplets. These data were obtained from the dataset of in situ observation during the increase in humidity. All the scale bars represent 300 nm.

Circular depressions, which are similar to nano-water droplets in size, were also observed on the smooth glass surface. These indentations appeared both with increasing humidity and after leaving the glass overnight at high humidity levels (circles in Figure S8). Unlike the nano-water droplets, these depressions exhibited a depth of 3–4 nm and did not show any evidence of diffusion. Similar to the nano-water droplets, these indentations disappeared after drying. Furthermore, as described before, the grooves consistently encircled the nano-water droplets. These grooves, a few nanometers deep, exhibited concavity from the base of the nano-water droplets toward the substrate side (Fig. 4a–d). Notably, at higher humidity levels (> 70%), some of these nano-water droplets formed a raised bank-like structure around the grooves (Fig. 4e,f). These findings suggest the likely co-existence of a flexible film, such as a nano-water film^10,17,18^, on the glass surface alongside the nano-water droplets. Despite experimental conditions being similar to those employed in our study, the wetting measurements of a silica glass using SFG spectroscopy revealed the absence of a homogeneous nano-water film at low humidity levels (~ 50%)^12^. This implies that the observed thin film may not have been purely composed of water.

Previous investigations focusing on the chemical alterations of oxidized silicon surfaces in moist air or water have reported the emergence of a Si–OH network near the substrate surface in response to adsorbed water^19–21^. This phenomenon leads to the formation of a silica gel-like layer with a thickness of a few nanometers (we named nano-liquid film). It is likely that the gel-like structure could not detected by SFG spectroscopy because the water molecules are unlikely to be polarized at solid–liquid and gas–liquid interfaces.

### Adhesion force mapping on silica glass surface by PFT

To validate the existence of the nano-liquid film (denoting a water thin film containing a silica gel layer) and delineate the distinctive physical properties between the nano-water droplets and the nano-liquid film, we investigated the wetting behavior of the silica glass surface using the peak force tapping (PFT) method. Adhesion force measurements were conducted within an area of 2 × 2 μm^2^ on the glass surface while gradually increasing the humidity within a closed cell. Note that the adhesion force varies among individual probes due to differences in the oxidation state of the tip surface and variations in tip shape. Therefore, we evaluated the changes in adhesion force by comparing data obtained using the same probe.

**Figure 5:**
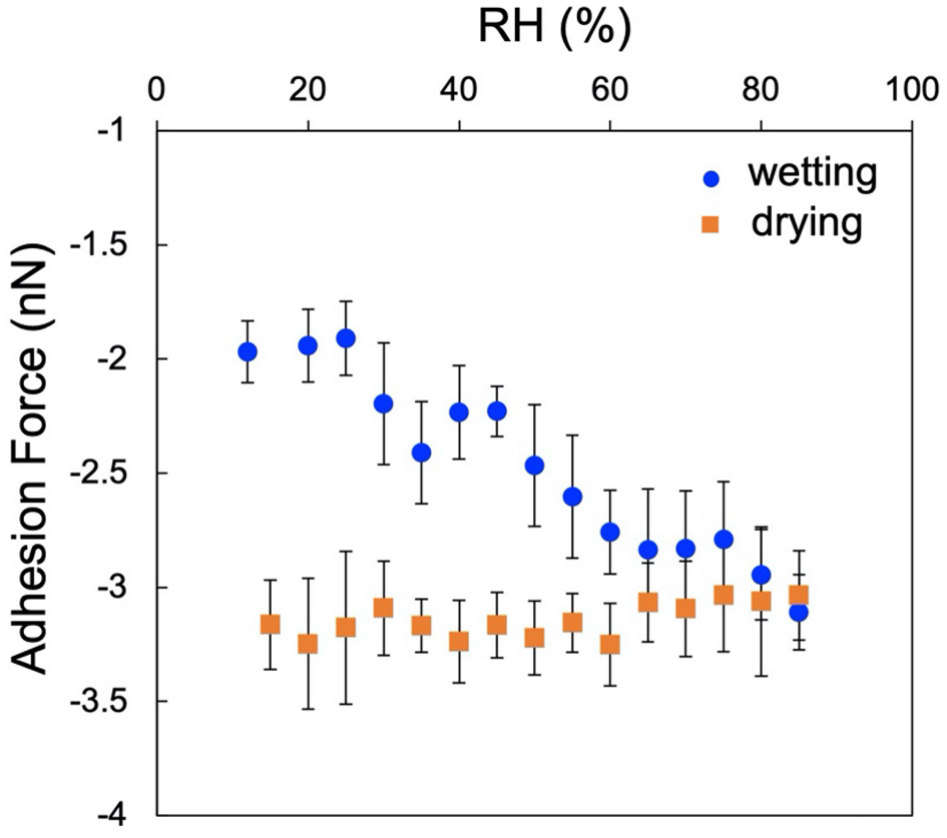
Change in average adhesion force on the flat surface of the glass with humidity by PFT measurements. Blue circle and square orange plots represent those measured during the process of increasing humidity (wetting) and decreasing humidity (drying), respectively. These adhesion forces were obtained by averaging the measurements at 10 points within a 2 μm scan area at each humidity from the dataset of Fig. 6.

When investigating the adhesion force of ten randomly selected areas devoid of nano-water droplets, the observations revealed a consistent increase in the adhesion force across the entire glass surface in response to the humidity, indicative of liquid film formation (Blue plots in Fig. 5). The exact thickness of this film remains undetermined; nevertheless, previous studies on the adsorption of mica and water on glass surfaces in high-humidity environments have suggested a thickness of several nanometers. Notably, even upon reducing the humidity, we found that the overall surface adhesion did not revert to its original state (Orange plots in Fig. 5), revealing that similar to the difficulty in desorbing adsorbed water from various solid samples^22^, the formation of a nano-liquid film on a silica glass surface is irreversible.

**Figure 6:**
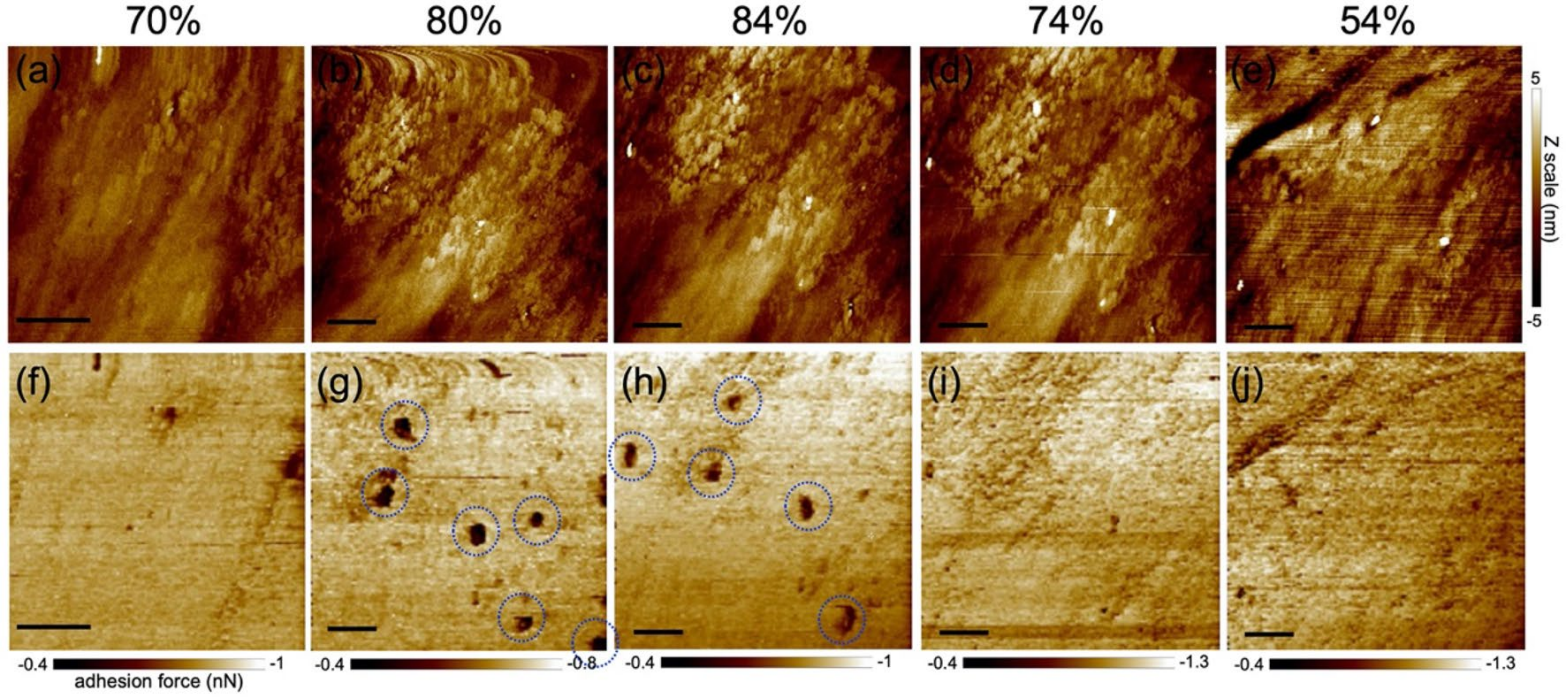
The changes in height images (256 pixels) (**a**–**e**) and adhesion maps (128 pixels) (**f**–**j**) with humidity observed by PFT mode. Peak force set point was 0.5 nN. In (**e**) and (**j**), the scan area was shifted due to thermal drift while dry air was brown in. Dotted circles in (**g**) and (**h**) indicate lower adhesion area. All scale bars represent 500 nm (scan area of (**a**) and (**f**) is smaller than the other images).

**Figure 7:**
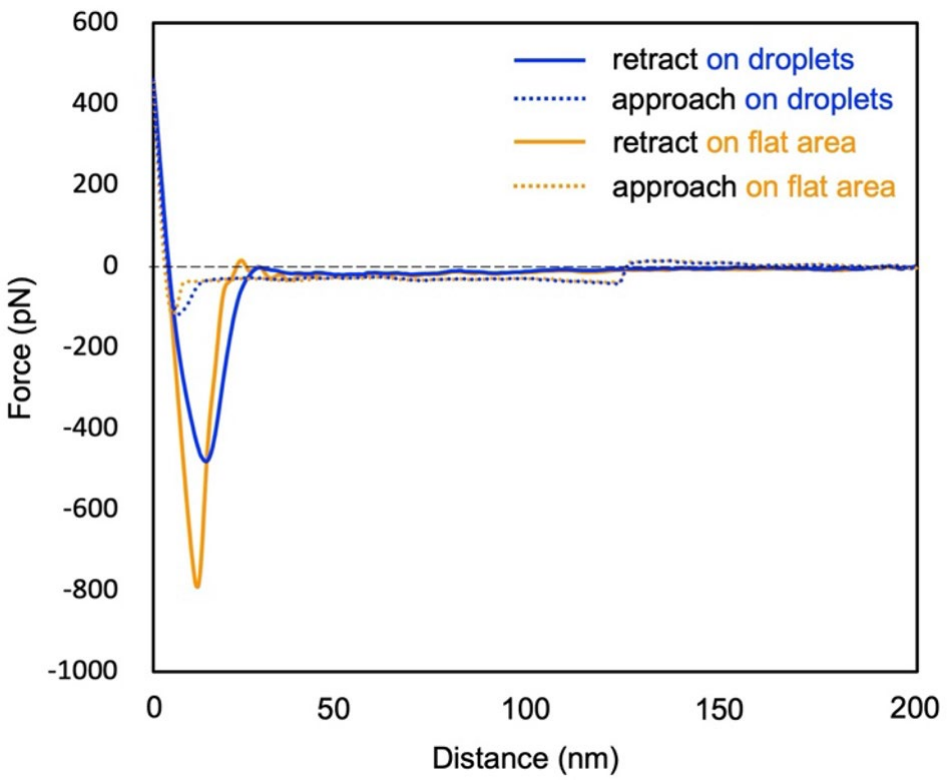
Force curves obtained from peak force tapping measurement at 80% RH. The average of 10 force curves acquired on nano water droplets (blue) and on flat area (orange), respectively. The dotted curves and the solid curves represent approach and retract curves, respectively.

When the humidity approaches 80%, distinct dark regions appeared in the adhesion map (Fig. 6), indicating areas of lower viscosity compared with their surroundings. Brighter regions in the map correlated with a higher adhesion force (indicating a higher viscosity). In the force curves obtained on bright and dark regions, a significant difference was observed in the minimum values in the retract curve, indicating adhesion force, whereas little difference observed in the approach curves between the dark and bright regions (Fig. 7). Comparing the average values of force curves obtained at 10 different locations, the adhesion force was found to be − 475 pN in the dark region and − 789 pN in the bright region, indicating a greater adhesion force on the bright region.

It is worth noting that external forces such as capillary forces derived from water surface tension are included in the force curve, so it does not strictly evaluate the viscosity of water on the glass surface. Nevertheless, it became evident that brighter and darker regions exhibit distinct physical properties. In addition, the measurement of the energy dissipation by the cantilever excitation signal in FM–AFM produced an image that reflects the viscosity of the sample^23,24^. The energy-dissipation image obtained in this study also suggests reduced dissipation (lower viscosity) within the nano-water droplet area, consistent with the PFT-mode results (Figure S9). The energy dissipation in the circular depressions remained nearly identical to that in the surrounding flat area, indicating that the structure and physical properties of the liquid film remained unchanged even within its depression region. The adhesion map revealed approximately five distinct low-adhesion regions within an area of 2 × 2 μm^2^, persisting steadily at humidity levels exceeding 80% and disappearing at an RH of approximately 70% upon humidity reduction.

The surface topographic images, along with the adhesion map, simultaneously acquired in the PFT mode did not show any nano-water droplets. This is attributed to the operational nature of the PFT mode, wherein the tapping motion of the probe while scanning the sample surface distorts the nano-water droplets, hindering their observation during scanning. Notably, some of the nano-water droplets evident from the adhesion map were located on the adsorbates observed in the topographic images. These observations support the FM–AFM findings, indicating that some of the droplets were formed at the adsorbate sites. The adhesion maps showed that the nano-water droplets exhibited a lower viscosity than the liquid film. The nano-water droplets were assumed to exhibit physical properties akin to those of bulk water because of the transportation of water molecules between the nano-water droplets and because they evaporate under dry conditions. Consequently, the result indicating the relatively lower viscosity of nano-water droplets relative to their surroundings implies that the nano-liquid film exhibited a higher viscosity than water. Structural water at the solid–liquid interface showed a higher viscosity than bulk water^24,25^. However, in this scenario, the water molecules will be polarized at the interface and should be detectable through SFG spectroscopy measurements. This outcome corroborates the formation of silica gel-like layers on the silica glass surfaces, as previously described.

### Surface/interfacial structure of nano-water droplets

Sessile droplets 100 nm in size have been reported in previous studies, in which distilled water was sprayed onto a metal substrate using air-blasting for observations^26,27^. These studies confirmed that nanometer-sized sessile droplets can exist stably at a humidity of approximately 50%. In contrast, our study demonstrated the spontaneous formation of nano-water droplets in humid environments. Despite the fundamental differences between sessile droplets and nano-water droplets in terms of their origin, it is necessary to consider the influence of organic impurities, as highlighted in a previous study. In their study, X-ray photoelectron spectroscopy (XPS) measurements showed the presence of an organic film on the surface of a water film that adhered to the metal substrate before the injection of nanodroplets. In our case, although the possibility of an organic film on the liquid film and the nano-water droplets cannot be completely dismissed, the contact angle of the sessile droplets on the organic film was 10°, whereas the estimated contact angle of the nano-water droplets, calculated from their radius and height^28^, was approximately 2°. This is similar to the wetting angle of macro water droplets on a clean glass surface^29^, indicating that an organic film is less likely to exist under the conditions employed in this study.

While the nano-water droplets were found to exhibit properties akin to those of bulk water, the repulsion between the droplets observed in this study suggests a unique interface structure. Although macroscopic water droplets can repel each other, this phenomenon typically occurs under conditions of substantial kinetic energy, such as when a droplet falls from a significant height onto the water surface^30^, which is unlikely in a static environment such as the one employed in this study. Moreover, in the absence of an organic film due to the previously mentioned reasons, a robust hydrogen bonding network is likely to exist at the air–liquid interface of the nano-water droplets. FM-AFM has been proven successful in observing the structure of liquids at the solid–liquid interface^31–33^, promising potential insights into elucidating the air–liquid interface structure of nanoscale water droplets in future research.

## Conclusions

High-resolution AFM observations of the wetting process on a silica glass surface revealed a spontaneous formation of nano-water droplets, depending on the humidity. In contrast to a previously proposed model, the nano-water droplets were found to not only form directly on the silica glass surface but also above the silica gel-like layer generated by the adsorbed water film at low humidity. This suggests a two-stage formation of the water film and nano-water droplets on solid surfaces dissolved in water. This implies that similar phenomena can occur not only on silica glass but also on other materials such as metals that form hydroxide layers or inorganic salts prone to deliquescence.

Furthermore, through in situ observations, the unique behaviors of nano-water droplets, such as surface diffusion and repulsion between droplets, were discovered, indicating the existence of an interface structure distinct from that seen in macroscopic water droplets. Although it is difficult to remove a water film from a material surface, it has been found that nano-water droplets undergo reversible evaporation and condensation in response to changes in humidity. Leveraging this property could offer possibilities for utilizing nano-water droplets as controllable adsorbates, potentially enhancing surface characteristics, such as friction control in materials. Additionally, the mobility of the nano-water droplets implies their potential to induce the transport of adsorbed substances on wet solid surfaces. Such microscopic behavior of nano-water droplets may contribute to the removal contaminants from material surfaces or the catalytic reactions on solid surfaces, such as the facilitation of organic material diffusion on mineral surfaces.

### Supplementary Information

Supplementary Figures.

## Data Availability

The datasets used and/or analyzed during the current study are available from the corresponding author upon request.
