# Supplementary material for: Microscopic behavior of nano-water droplets on a silica glass surface
Source: Sci Rep. 2024 May 10;14:10693. doi: 10.1038/s41598-024-61212-1 (PMC11082177; doi:10.1038/s41598-024-61212-1)
Supplement: Supplementary file 1 — Supplementary Figures. [file 41598_2024_61212_MOESM1_ESM.docx]

**Supplementary**

**Microscopic behavior of nano-water droplets on a silica glass surface**

Yuki Araki^*^, Taketoshi Minato, Toyoko Arai


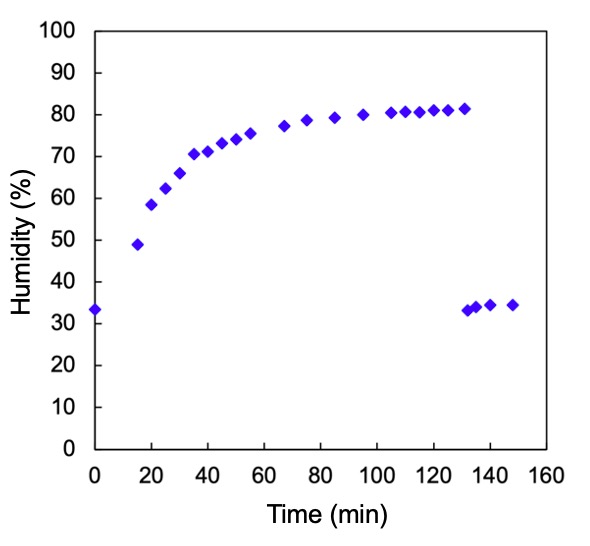


**Figure S1** Humidity variation in the incubator during FM-AFM imaging.


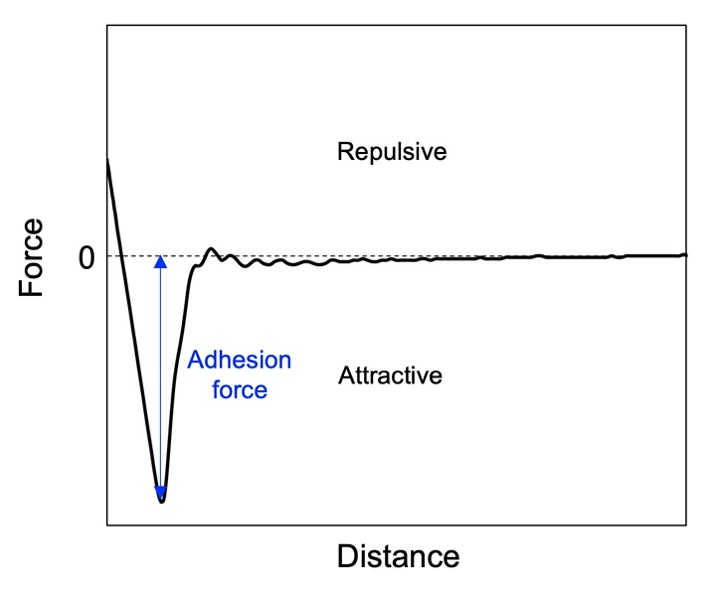


**Figure S2** Typical retract curve obtained in PFT measurement. Blue arrow indicates the values measured as adhesion force.


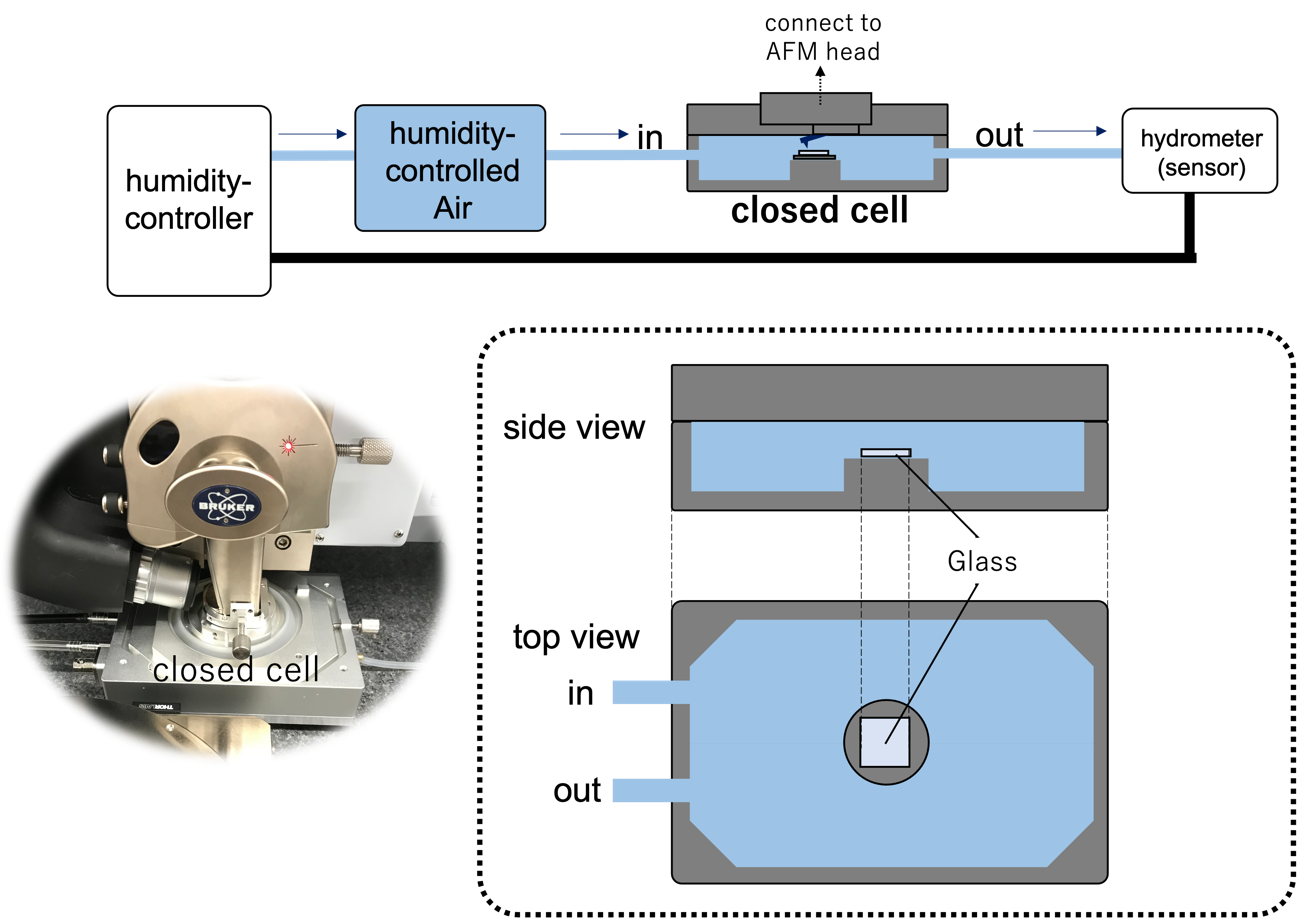


**Figure S3** Schematic of humidity control system (upper image) and an enlarged image of the closed cell (bottom image) for peak force tapping (PFT) measurement.


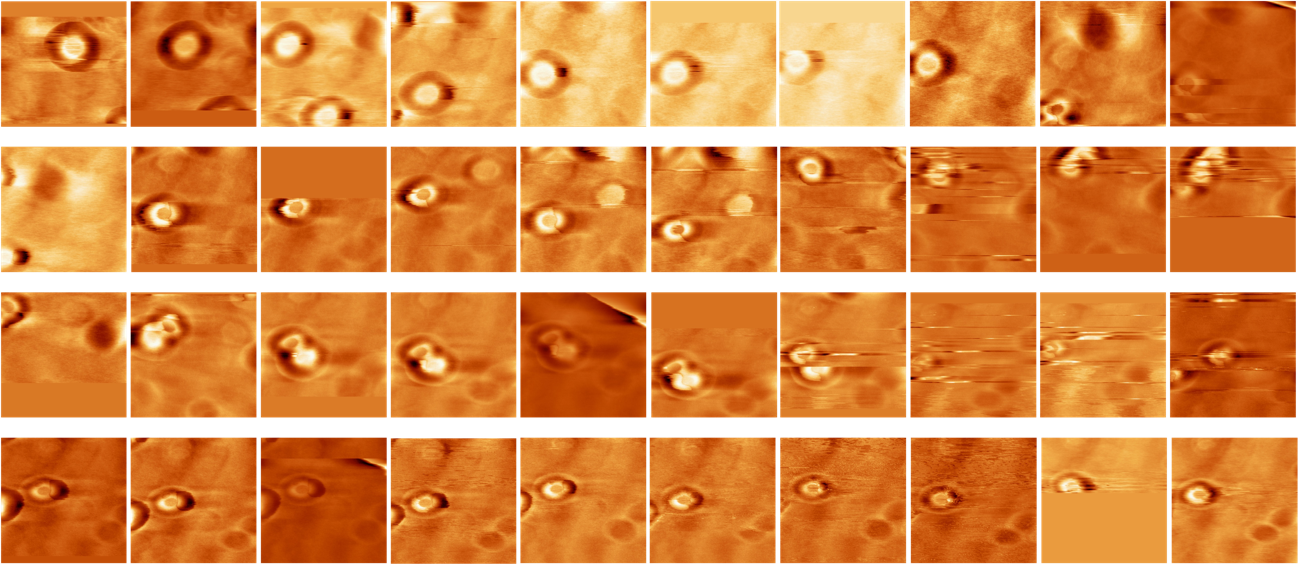


**Figure S4** Continuous images of the topography of a silica glass surface in humidification process in the same scan area observed for 90 min by FM–AFM. The relative humidity varied from 50% to 80%, between the first and final images. All the images are observed in 1.5 × 1.5 μm^2^.


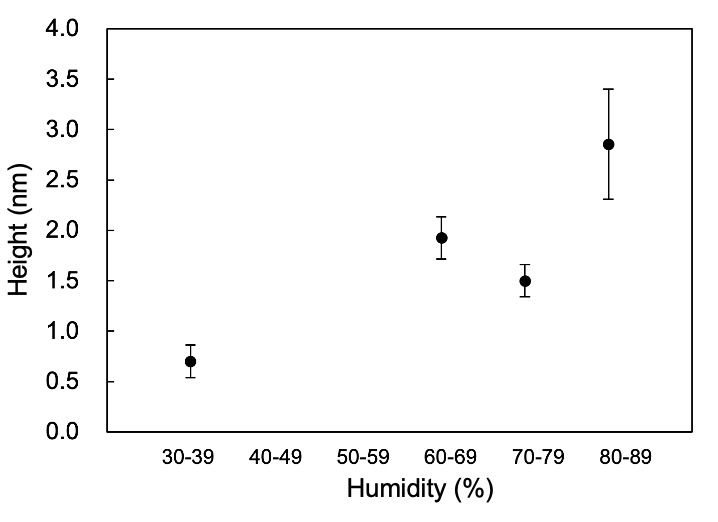


**Figure S5** Variation in the average height of nano-water droplets across different humidity levels.


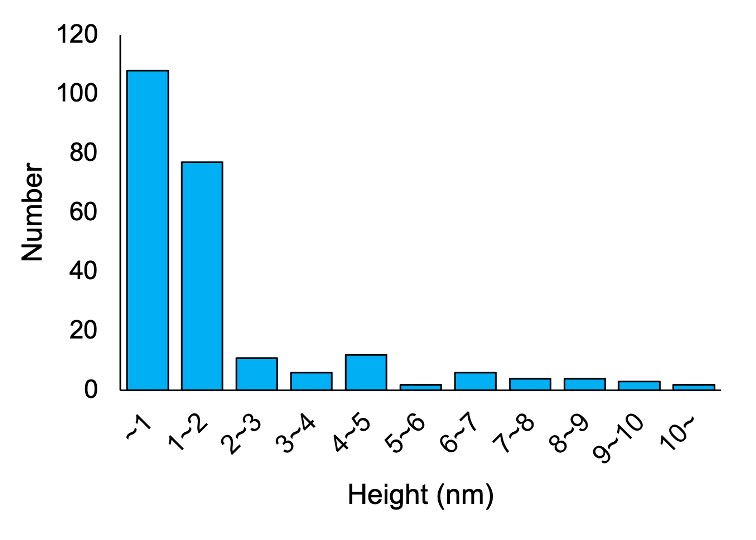


**Figure S6** Height distribution of the nano-water droplets on a silica glass at 85% RH.


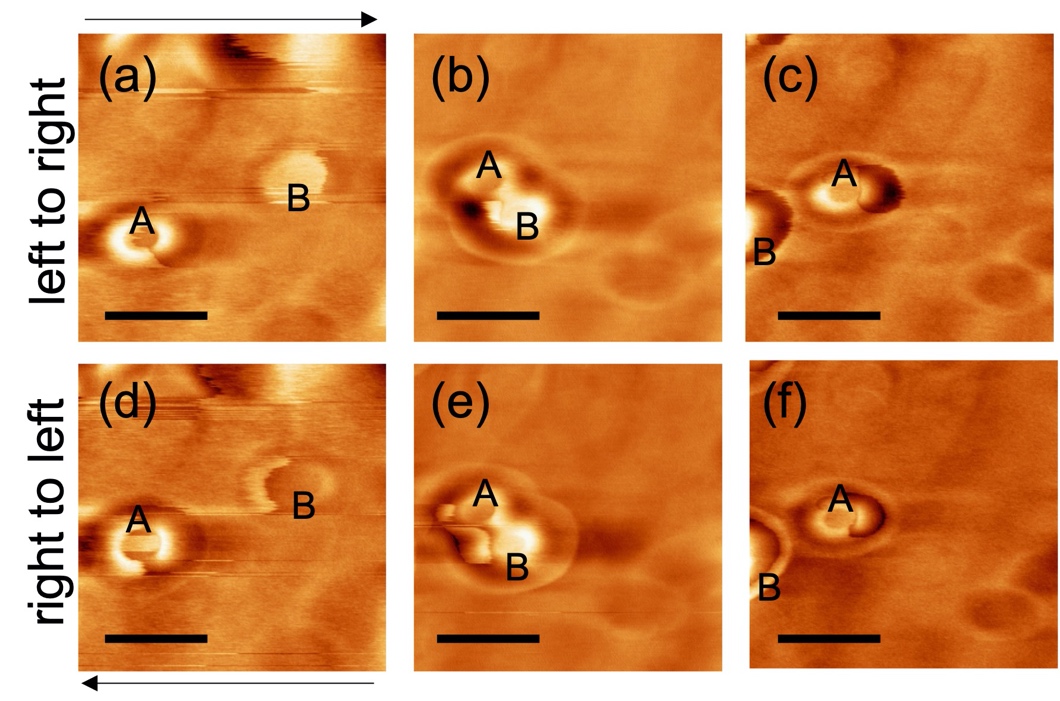


**Figure S7** Comparison of the diffusion direction of nano water droplets with the scanning direction. (a)~(c) depict surface topography images identical to Figure 2(b)~(d), acquired by scanning from left to right. (d)~(f) show images obtained in the opposite scanning direction. Arrows in the figure represent the respective scanning directions. All scale bars represent 500 nm.


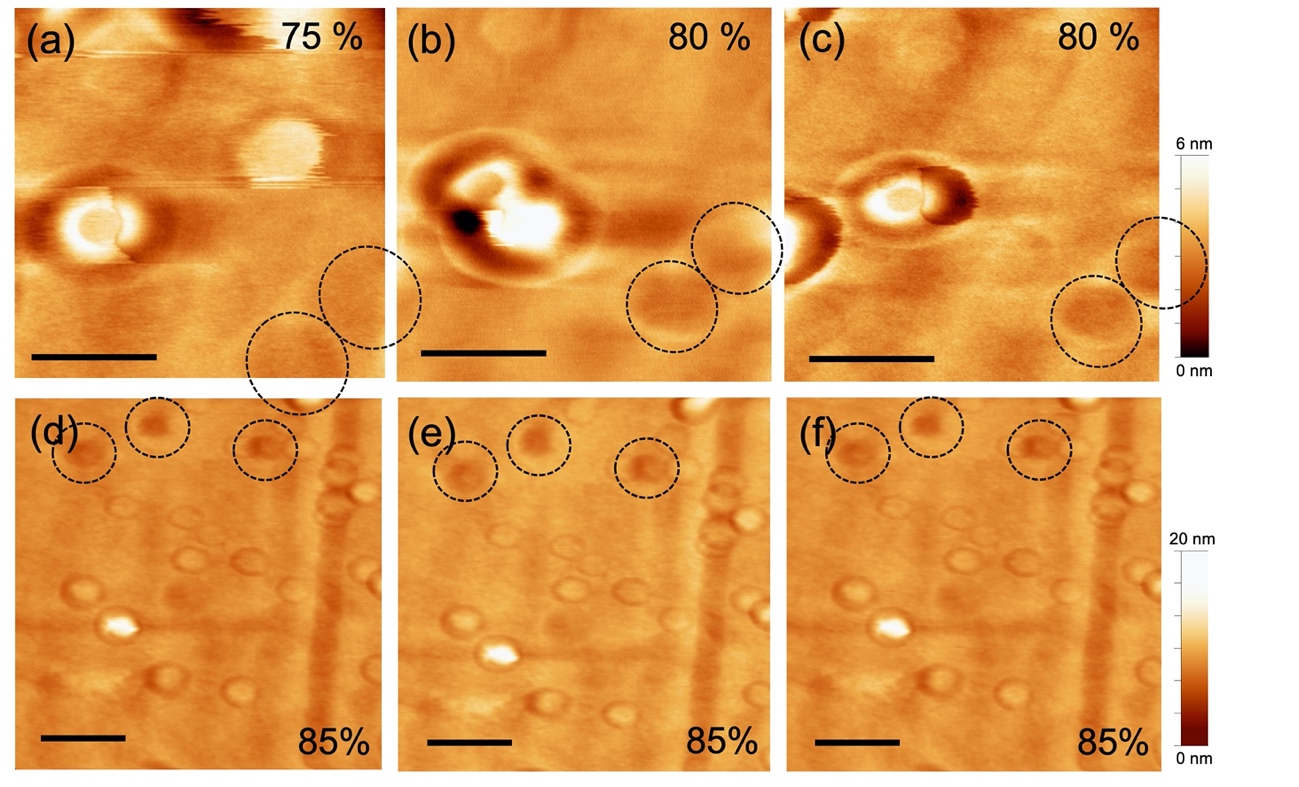


**Fig.S8** Circular depressions observed in topographic image obtained by FM-AFM. Dashed circles indicate circular depressions. (a)~(c) and (d)~(f) are the same image as Figure 2(b)~(d) and Figure 3(a)~(c), respectively. All scale bars represent 500 nm.


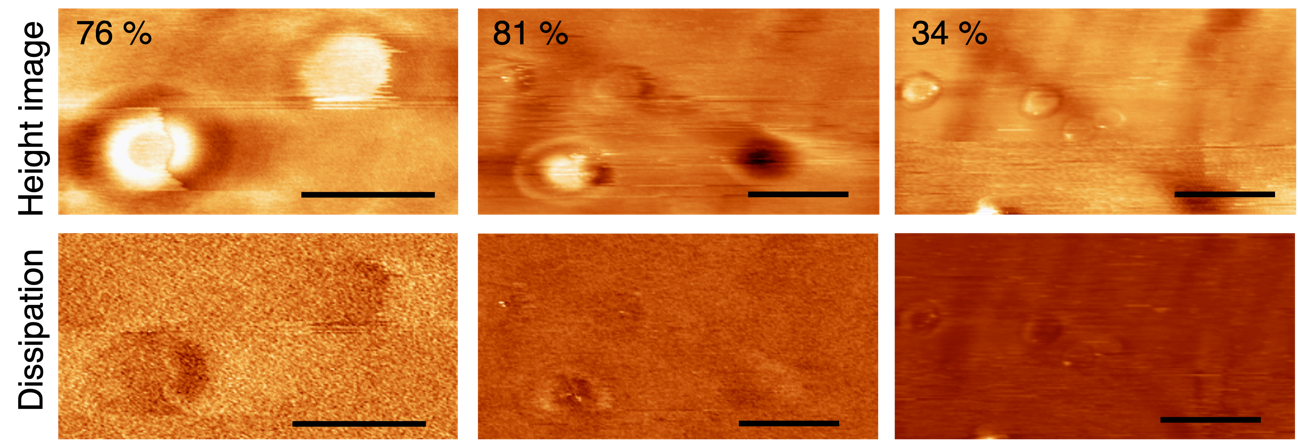


**Figure S9** Dissipation images of nano-water droplets at various humidity levels obtained by FM–AFM. The upper images are the topographic images, and the lower images are the simultaneously acquired energy dissipation images. All the scale bars represent 500 nm.
